# Analysis of short tandem repeats linked to polyglutamine diseases from whole-genome sequencing reveals intermediate alleles of *HTT* associated with an early disease onset in *C9orf72* carriers

**DOI:** 10.1093/braincomms/fcaf220

**Published:** 2025-06-04

**Authors:** Mathieu Barbier, Thomas Gareau, Agnès Camuzat, Marine Guillaud-Bataille, Susana Boluda, Fabienne Clot, Lara Araktingi, Barbara Borroni, Julie van der Zee, Roberta Ghidoni, Sonia Bellini, Daniela Galimberti, Giacomina Rossi, Benedetta Nacmias, Beatriz De la Casa-Fages, Pau Pastor, French Clinical and Genetic Research Network on FTD/FTD-ALS and PrevDemALS Study Groups, Alexis Brice, Sophie Auriacombe, Serge Belliard, Frédéric Blanc, Stéphanie Bombois, Claire Boutoleau-Bretonnière, Agnès Camuzat, Mathieu Ceccaldi, Philippe Couratier, Vincent Deramecourt, Mira Didic, Frédérique Etcharry-Bouyx, Maïté Formaglio, Véronique Golfier, Didier Hannequin, Lucette Lacomblez, Julien Lagarde, Isabelle Le Ber, Richard Levy, Florence Pasquier, Thibaud Lebouvier, Carole Roué-Jagot, Anne Salmon, Marie Sarazin, Christel Thauvin-Robinet, Catherine Thomas-Anterion, Jérémie Pariente, François Sellal, Daisy Rinaldi, Adeline Rollin-Sillaire, Martine Vercelletto, David Wallon, Morwena Latouche, Eric le Guern, Alexandra Durr, Annie Laquerrière, Rob Moccia, Danielle Seilhean, Victoria Alvarez, Isabelle Le Ber

**Affiliations:** Sorbonne Université, Paris Brain Institute—Institut du Cerveau, ICM, Inserm U1127, CNRS UMR 7225 APHP—Hôpital Pitié-Salpêtrière, Paris 75013, France; Sorbonne Université, Paris Brain Institute—Institut du Cerveau, ICM, Inserm U1127, CNRS UMR 7225 APHP—Hôpital Pitié-Salpêtrière, Paris 75013, France; Sorbonne Université, Paris Brain Institute—Institut du Cerveau, ICM, Inserm U1127, CNRS UMR 7225 APHP—Hôpital Pitié-Salpêtrière, Paris 75013, France; Department of Medical Genetics, AP-HP Sorbonne Université, UF de Neurogénétique Moléculaire et Cellulaire, Hôpital Pitié-Salpêtrière, Paris 75013, France; Sorbonne Université, Paris Brain Institute—Institut du Cerveau, ICM, Inserm U1127, CNRS UMR 7225 APHP—Hôpital Pitié-Salpêtrière, Paris 75013, France; Department of Neuropathology, APHP—Hôpital de la Pitié-Salpêtrière, Sorbonne Université, Paris 75013, France; Department of Medical Genetics, AP-HP Sorbonne Université, UF de Neurogénétique Moléculaire et Cellulaire, Hôpital Pitié-Salpêtrière, Paris 75013, France; Sorbonne Université, Paris Brain Institute—Institut du Cerveau, ICM, Inserm U1127, CNRS UMR 7225 APHP—Hôpital Pitié-Salpêtrière, Paris 75013, France; Department of Clinical and Experimental Sciences, Centre for Neurodegenerative Disorders, University of Brescia, Brescia 25100, Italy; Molecular Markers Laboratory, IRCCS Istituto Centro San Giovanni di Dio Fatebenefratelli, Brescia 25100, Italy; Neurodegenerative Brain Diseases, VIB-UAntwerp Center for Molecular Neurology, VIB, Antwerp B-2610, Belgium; Department of Biomedical Sciences, University of Antwerp, Antwerp 2000, Belgium; Molecular Markers Laboratory, IRCCS Istituto Centro San Giovanni di Dio Fatebenefratelli, Brescia 25100, Italy; Molecular Markers Laboratory, IRCCS Istituto Centro San Giovanni di Dio Fatebenefratelli, Brescia 25100, Italy; Department of Biomedical, Surgical and Dental Sciences, University of Milan, Milan 20122, Italy; Fondazione IRCCS Ca’ Granda, Ospedale Maggiore Policlinico, Milan 20122, Italy; Unit of Neurology V and Neuropathology, Fondazione IRCCS Istituto Neurologico Carlo Besta, Milan 20133, Italy; Department of Neuroscience, Psychology, Drug Research and Child Health University of Florence Azienda Ospedaliero-Universitaria CareggiViale, Florence 50100, Italy; IRCCS Fondazione Don Carlo Gnocchi, Florence 50100, Italy; Neurology Department, Movement Disorders Unit, Hospital General Universitario Gregorio Maranon, Madrid 28007, Spain; Department of Neurology, Unit of Neurodegenerative Diseases, University Hospital Germans Trias i Pujol and The Germans Trias i Pujol Research Institute (IGTP) Badalona, Barcelona 08916, Spain; Sorbonne Université, Paris Brain Institute—Institut du Cerveau, ICM, Inserm U1127, CNRS UMR 7225 APHP—Hôpital Pitié-Salpêtrière, Paris 75013, France; PSL Research University, EPHE, Paris 75014, France; Department of Medical Genetics, AP-HP Sorbonne Université, UF de Neurogénétique Moléculaire et Cellulaire, Hôpital Pitié-Salpêtrière, Paris 75013, France; Sorbonne Université, Paris Brain Institute—Institut du Cerveau, ICM, Inserm U1127, CNRS UMR 7225 APHP—Hôpital Pitié-Salpêtrière, Paris 75013, France; Department of Pathology, Normandie Université, INSERM U1245, Rouen University Hospital, Rouen 76000, France; Rare Disease Research Unit, Pfizer Inc., Cambridge, MA 02139, USA; Sorbonne Université, Paris Brain Institute—Institut du Cerveau, ICM, Inserm U1127, CNRS UMR 7225 APHP—Hôpital Pitié-Salpêtrière, Paris 75013, France; Department of Neuropathology, APHP—Hôpital de la Pitié-Salpêtrière, Sorbonne Université, Paris 75013, France; Laboratory of Genetics, Hospital Universitario Central de Asturias, Oviedo 33011, Spain; Instituto de Investigación Sanitaria del Principado de Asturias (ISPA), Oviedo 33011, Spain; Sorbonne Université, Paris Brain Institute—Institut du Cerveau, ICM, Inserm U1127, CNRS UMR 7225 APHP—Hôpital Pitié-Salpêtrière, DMU Neuroscience, Paris 75013, France; Département de Neurologie, Center for Rare or Early-Onset Dementias, IM2A, AP-HP-Hôpital Pitié-Salpêtrière, Paris 75013, France

**Keywords:** C9orf72, frontotemporal dementia, amyotrophic lateral sclerosis, huntingtin, whole-genome sequencing

## Abstract

Carriers of the GGGGCC pathogenic expansion in *C9orf72* can develop symptoms of frontotemporal dementia and/or amyotrophic lateral sclerosis, with variable and unpredictable ages at onset. Previous studies aiming to decipher the genetic bases of the clinical variability in this rare disease included bi-allelic polymorphisms, excluding short tandem repeats. Whole-genome sequencing data of 195 *C9orf72* patients were used to consider all short tandem repeats linked to polyglutamine disorders as potential genetic modifiers given the existing links between *C9orf72* and polyglutamine diseases. Intermediate alleles of *HTT* encoding huntingtin were associated with an earlier age at onset among *C9orf72* carriers in the discovery cohort (*n* = 195, *P* = 0.0003) and in a European replication cohort (*n* = 145, *P* = 0.006). In the merged cohort (*n* = 340), the average difference of age at disease onset was 9.42 ± 2.14 years (*P* = 1.3 × 10^e−5^) between carriers and non-carriers of *HTT-*intermediate alleles. Neuropathology of one *C9orf72* case heterozygous for *HTT-*intermediate allele showed typical TDP-43 inclusions related to the *C9orf72* pathogenic expansion and was negative for polyglutamine inclusion. No somatic expansion of *HTT* was detected in blood of all *C9orf72exp*/*HTT-*intermediate carriers. If this study reinforces potential biological links between huntingtin and C9orf72 that remain to be explored, the results also illustrate the interest of considering short tandem repeats from whole-genome data in association studies which paves the way to more exhaustive approaches to explore the trait heritability due to short-tandem-repeats still hidden in the genome.

## Teaser

Whole-genome-sequencing of patients carrying pathogenic *C9orf72* expansions was used to consider short-tandem-repeats linked to polyglutamine disorders as potential modifiers of frontotemporal-dementia/amyotrophic-lateral-sclerosis. Barbier *et al*. reported intermediate alleles of huntingtin associated with early disease onsets, highlighting links between *C9orf72* and polyglutamine disorders, and emphasizing the role of short-tandem-repeats in disease variability.

## Graphical Abstract

**Graphical Abstract:**
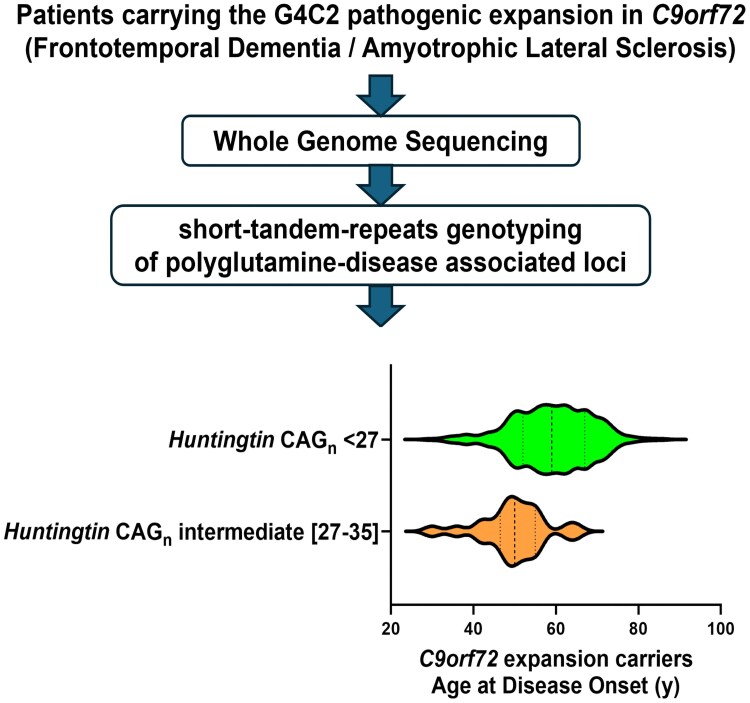

## Introduction

A GGGGCC pathogenic expansion in the *C9orf72* gene is the main genetic cause of frontotemporal dementia (FTD) and amyotrophic lateral sclerosis (ALS).^1,2^ Since its discovery, intriguing links appeared between this GGGGCC expansion and polyglutamine (polyQ) disorders due to exonic CAG expansions, leading to Huntington disease (HD) and several spinocerebellar ataxias. The GGGGCC repeat expansion triggers mislocalization and inclusions of TAR DNA-binding protein 43 (TDP-43) in *C9orf72* expansion carriers^3^ while polyglutamine (polyQ) inclusions are hallmarks of CAG expansion diseases. Despite this dichotomy, some works reported co-occurrence of polyglutamine and TDP-43 inclusions in HD patients’ brain.^4-6^ Although it is still discussed if huntingtin (HTT) and TDP-43 co-localize within inclusions, these studies confirmed the widespread presence of both types of inclusions at all HD stages. TDP-43 was previously found to interact with HTT carrying pathogenic CAG repeats.^7^ In addition, it has recently been shown that *ATXN1* and *ATXN2* influence the nucleocytoplasmic ratio of TDP-43, and that CAG tracks stimulate the cytoplasmic mis-localization of TDP-43 preceding aggregates.^8-10^ Furthermore, *ATXN2* is a genetic modifier of *C9orf72*-disease, intermediate CAG repeats being associated with the risk to develop ALS.^11^

The pioneer example of *ATXN2* illustrates how short tandem repeats (STRs) can influence other disease outcomes even in the physiological range of repeat number. STRs account for ∼3% of the human genome, are highly polymorphic, but are totally ignored in genome-wide association studies including in *C9orf72*-disease-related genome-wide association studies.^12-14^ In some cases, the variability in repeat numbers has a functional impact leading to so-named ‘expansion diseases’ when the number of repeats exceeds a certain threshold,^15^ reinforcing the interest of implementing this type of functional polymorphisms in association studies. Recently developed bioinformatic tools allow a detection of pathogenic STRs and overall, a robust sizing of repeat number from short-read whole-genome data especially when the repeat sizes are in the range of short-reads like normal or intermediate alleles (IA) of polyQ loci.^16,17^

Here, we take advantage of the whole-genome sequencing of 195 *C9orf72* patients to perform the analysis of CAG repeats in all loci linked to polyQ disorders as potential disease modifiers given the clinical, neuropathological and genetic links between *C9orf72* and polyQ diseases.

## Materials and methods

### Patients

Patients of the discovery cohort (starting *n* = 200 before the QC performed on whole-genome data) were selected from a large cohort of 590 *C9orf72* patients from European origin (mostly French) from 424 families including index cases and relatives. They were enrolled by expert neurologists of a national research network on FTLD/FTLD-ALS (project #RBM02-59) and PREVDEMALS study group.^18^ Age at onset of affected subjects were reviewed by two evaluators based on patient’s clinical charts and on caregiver’s interviews, as previously described.^18^ Age at onset of FTD was defined as the age of occurrence of the first symptom (either behavioural, language or motor). In most cases, a second informant was questioned to accurately determine the disease onset. The presence of ALS symptoms at the most recent examination was also noted, as well as the age at ALS onset self-reported by patients of second informants. Patients with discordant data for the age at onset between sources of information, low-quality or unavailable DNA were excluded. The age at death (and subsequently the disease duration at death) was also included as phenotypes when available (*n* = 58). All participants were included in research studies after written informed consent obtained from the patients or their guardians, in agreement with their national bioethic laws (IRB: CPP Ile de France II, project #RBM 02-59).

Patients of the replication cohort are from European origin. All information regarding recruitment, countries of origin, diagnoses criteria, medical records and ethical consideration have been previously published.^19^

Only unrelated patients with both *HTT* CAG repeat number and age at disease onset available were included in the present study as the replication cohort (*n* = 145). Both sex ratio and mean age at onset were similar between the discovery and replication cohorts. Main details about patients’ cohorts used in this work are shown in Table 1.

**Table 1:** Description of cohorts

	Discovery	Replication	Merged
*N*	195	145	340
Males (%)	110 (56)	77 (53)	187 (55)
FTD	120	117	237
FTD-ALS	61	28	89
ALS	14		14
Mean AO ± SD (years)	58.83 ± 9.76	58.18 ± 9.77	58.55 ± 9.75
[min–max] AO (years)	[30–86]	[29–79]	[29–86]

In order to analyse potential correlations between GGGGCC repeat number in *C9orf72* and CAG repeat number, we gathered *C9orf72* carriers for whom GGGGCC repeat number was previously estimated using southern blots and also included in the current discovery cohort (*n* = 30). Pre-symptomatic *C9orf72* carriers (*n* = 30) for whom the GGGGCC repeat number has been also previously established using southern blots^20^ were also included to enlarge as much as possible the number of individuals for comparisons between GGGGCC and CAG repeat numbers.

### Whole-genome short read sequencing

Whole-genome library preparations were realized following manufacturer’s recommendations (DNA prep from ILLUMINA). Final samples pooled library preps were sequenced on Novaseq 6000 ILLUMINA with S4-300 cycles cartridge (2×10000Millions of 150 bases reads), corresponding to 2×400Millions of reads per sample after demultiplexing at least. Genomic data processing, starting from raw FASTQ files, was conducted using the Illumina Dragen DNA pipeline v3.10. This encompassed adapter trimming, alignment to the hg38 reference genome and deduplication of PCR bias.

VCF files from whole-genome sequencing were used to perform a first SNP-based quality control per patient using PLINK v1.90b3w.^21^ Five individuals were excluded due to sex discordance (*n* = 2), detection of duplicates (*n* = 2) or unexpected relatedness (*n* = 1). Thus, 195 patients were kept for downstream analyses (Table 1). All remaining patients (*n* = 195) have <5% of missing SNP data, and the mean genotyping rate was >98% after this QC.

### Determination of CAG repeat number in polyQ loci from whole-genome sequencing

Expansion calling was executed through ExpansionHunter^22^ integrated into Dragen, leveraging a custom catalogue with specified polyQ loci, location and expected CAG motif. This simultaneous analysis from whole-genome data included the 10 loci described so far to be associated with polyQ diseases: *AR*, *ATN1*, *ATXN1*, *ATXN2*, *ATXN3*, *ATXN7*, *CACNA1A*, *HTT*, *JPH3* and *TBP*. The standard mode for consideration of off-targets was used to minimize overestimation of repeat numbers. Results include a genotype (number of repeats for short and long alleles) for each locus and each patient. Individual genotypes with a coverage <10× were excluded. The mean coverage for each expansion was comprised between 31.90 and 58.97. Genotype rates were >95% for all polyQ loci. Detailed information per locus can be found in Table 2. Consensus thresholds from GeneReviews (GeneReviews® - NCBI Bookshelf (nih.gov)) and literature were applied to classify repeat number as normal, intermediate (IA) or pathogenic alleles. Length and composition of repeats were visualized from BAM files using IGV and STRipy (STRipy - Short Tandem Repeats database and software) to search for somatic expansions or interruptions.

**Table 2:** Descriptive data of CAG repeats in polyglutamine disease-associated loci from whole-genome sequencing

Locus	CAG repeat coordinates (hg38)	Mean coverage (X)	Genotyping rate (%)	min–max CAG number	Carriers of IA
*AR*	chrX:67545316-67545385	31.9	99	8–35	0
*ATN1*	chr12:6936728-6936773	52.81	100	11–30	0
*ATXN1*	chr6:16327635-16327722	56.5	100	23–38	17
*ATXN2*	chr12:111598950-111599019	31.96	99	15–29	4
*ATXN3*	chr14:92071010-92071040	43.66	100	11–34	0
*ATXN7*	chr3:63912685-63912715	35.62	96	1–14	0
*CACNA1A*	chr19:13207858-13207897	35.57	100	4–15	0
*HTT*	chr4:3074876-3074933	39.54	100	9–34	11
*JPH3*	chr16:87604287-87604329	58.97	100	11–22	0
*TBP*	chr6:170561907-170562015	46.37	100	29–44	4

### PCR-based fragment length determination of *HTT* CAG repeat number in the discovery and replication cohorts

The genotyping of *HTT* CAG repeat number from whole-genome data using ExpansionHunter was previously shown to be robust.^16,17^ Nonetheless, the number of CAG repeats in exon 1 of *HTT* was determined in patients with *IA-HTT* using a PCR containing the repeat, followed by a fragment analysis as previously described.^23^ Repeat number estimates from whole-genome data were in full accordance with those from the PCR-based method (Supplementary Fig. 1). Measure of *HTT* CAG number in the replication cohort was also done using a PCR-based method as described elsewhere.^19^

### Neuropathology

The patient was part of the national Neuro-CEB brain donation program for research into neurodegenerative diseases. Post-mortem sampling was carried out according to the protocol used for the analysis of frontotemporal lobar degeneration. In available regions (including the frontal, temporal, parietal, occipital cortices, hippocampus, medulla oblongata, striatum and spinal cord), paraffin blocks were cut to a thickness of 3 μm. Immunohistochemistry studies were performed with the Ventana BenchMark XT stainers using antibodies against the following antigens: TDP-43 (PTG; polyclonal; 1/2000), phosphorylated TDP-43 (pTDP-43) (Cosmo Bio, pS409/410-2, polyclonal, 1/5000), polyglutamine repeats (1C2, monoclonal, Eurogenex; 1/4000), ubiquitin (Dako, polyclonal, 1/500), p62 (3/p62 LCK, Biosciences, 1/500) and monoclonal N-terminal HTT antibody MAB5492 (clone 2B4, Merck, 1/250) as described.^24,25^ Technical details can be found in Supplementary Table 1. The harmonized neuropathological classification was applied.^26^ One neuropathological HD case (harbouring 40 CAG for the longest allele in *HTT*) was used as a positive control for immunostaining as well as a patient without neurological disease at death (55 years) as a negative control. Additional information about the HD case can be found in Supplementary Table 2.

### Statistical analyses

As the age at onset followed a normal distribution in our cohort (passed normality tests), a Pearson *r* correlation test was used to correlate repeat numbers in polyQ loci with quantitative traits as a first approach. The repeat number of the two alleles was analysed separately (short and long alleles). Smoothing was obtained with five points in smoothing windows for all LOWESS curves designed to address complex patterns in scatterplot data.^27^ Mann–Whitney U-tests were used to compare the mean ages at onset between *IA-HTT* and *ATXN1-IA* carriers versus non-carriers. Means ± SEM are indicated.

We used a univariate mixed linear model to challenge the association between *IA-HTT* and quantitative traits with the genome-wide efficient mixed model association algorithm implemented in the software GEMMA v0.98.5.^28^ GEMMA fits a univariate linear mixed model to correct for population stratification, cryptic relationship and kinship. The kinship matrix was created with GEMMA. Genotypes for *TMEM106B* rs6966915, *C6orf10* rs9357140 and *SLITRK2* rs1009776 were extracted to be used as covariates in the univariate linear mixed models, also adjusted on sex and kinship. Results of the association test include beta coefficient (*β*), and adjusted *P*-value derived from the likelihood ratio test. The development (or not) of ALS symptoms at last examination was considered as a binary trait. Correlations between CAG repeat number and the development ALS symptoms for all loci were assayed using logistic regressions (*P*-value derived from the likelihood ratio test).

## Results

### Analyses of polyQ STRs repeat number and composition from whole-genome data in the discovery cohort

No pathogenic repeat in polyQ associated loci was detected among *C9orf72* carriers. CAG repeat numbers were not correlated with each other. IA were observed for (ranges of CAG repeat number and frequency of carriers in our cohort are indicated): *ATXN1* [36–38] *n* = 17 (8.7%), *ATXN2* [27–29] *n* = 4 (2%), *HTT* (CAG_n_) [27–34] *n* = 11 (5.6%), and *TBP* (CAG_n_ [40–44], *n* = 4 (2%). Table 2 resumes numerical data about polyQ STRs repeat number genotyped from whole-genome data in the discovery cohort. The frequency of IA was close to the frequency observed in super-populations from the gnomAD initiative except for *ATXN1* with a slightly higher frequency of IA carriers in our cohort (*P*_Chi-square_ = 0.028), however non-significant after correction for multiple testing. A careful examination of sequencing reads revealed interruptions in *ATXN1*, *ATXN2*, *HTT* and *TBP*. In *ATXN1*, the canonical CAG repeat including two CAT interruptions was found in the majority of individuals. Thirty-seven individuals had a loss of one or an additional CAT interruption. The CAA interruption was detected in *ATNX2* with 1–3 interruptions per patient (two interruptions being the most frequent motif observed). Also, a duplication of the CAACAG interruption at the 3′ end of the CAG track of *HTT* was detected in six patients. All these interruptions were already described and not associated with any of the clinical traits analysed.

The mean *C9orf72* repeat number was previously estimated using southern blots^20^ for a subset of patients included in the discovery cohort and in a cohort of *C9orf72* pre-symptomatic carriers allowing a larger comparison between GGGGCC and CAG repeat numbers in 60 individuals. There was no significant correlation between the *C9orf72* and CAG repeat numbers. Of note, the length of the *C9orf72* short (non-expanded) allele estimated from whole-genome data, was not associated with clinical traits.

### Association of polyQ CAG repeat number with clinical traits in the discovery cohort

**Figure 1:**
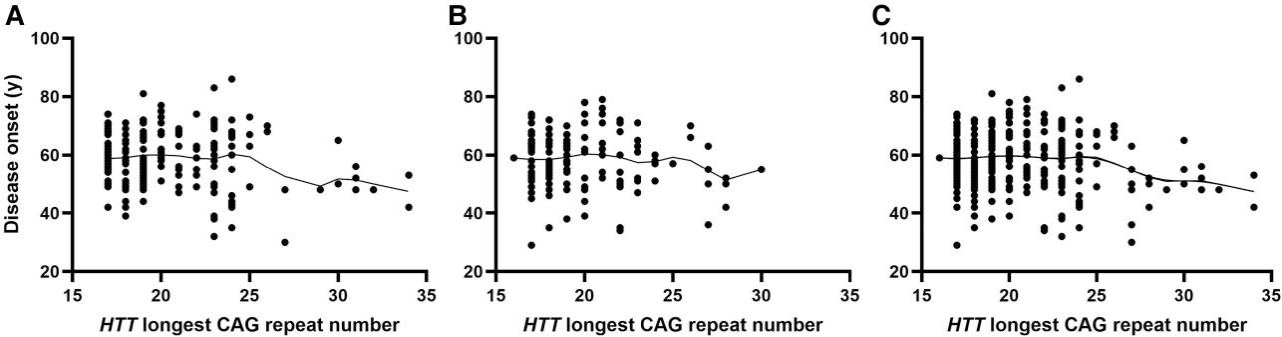
**Age at disease onset in function of *HTT* CAG repeat number (longest allele) in *C9orf72* patients.** (**A**) discovery cohort (*n* = 195); (**B**) replication cohort (*n* = 145); (**C**) merged cohort (*n* = 340). LOWESS curves are appearing for each graph. Each data point represents the longest number of CAG in *HTT* for each *C9orf72* patient.

The effect of each allele on phenotypes was tested separately (short and long alleles). A suggestive association was observed between CAG repeat number in *ATXN2* and presence of ALS symptoms (*P* = 0.075) and three out of four *ATXN2*-IA/*C9orf72exp* carriers developed symptoms of ALS, although the small number of carriers precluded statistical analyses. The longest values of CAG repeat number were significantly associated with the disease onset in (Table 3): *HTT* (*r* = −0.15, *P* = 0.034) and *ATXN3* (*r* = 0.17, *P* = 0.016). A separate analysis considering the age of onset of FTD or ALS led to the same trend of correlation in *HTT* (*r* = −0.16, *P* = 0.033 for FTD; *r* = −0.24, *P* = 0.049 for ALS) conversely to the *ATXN3* locus for which the correlation coefficients were not significant when considering the two phenotypes separately. The correlation profile between the disease onset and the longest CAG repeat number in *HTT* was not evocative of a linear effect (and not significant after correction for multiple testing) suggesting that a linear model may not be the appropriate model to fit with the observed data (Fig. 1A). By contrast, the LOWESS curve suggested of a threshold effect starting from CAG_n_ = 27 to 34, the longest repeat number detected in our cohorts. Interestingly, this range of CAG_n_ belongs to the range of IA of *HTT* [27–35]. Furthermore, the association between CAG repeat number in *HTT* and the disease onset completely disappeared after having removed *IA-HTT* carriers from the analysis (*r* = 0.021, *P* = 0.79). This supported the interest of comparing *C9orf72* patients with or without IA. Besides, no signal of associations was detected with other traits tested, also when considering shortest alleles of each polyQ locus.

**Table 3:** Analyses of CAG repeat number (longest alleles) with clinical traits in the discovery cohort

	*AR*	*ATN1*	*ATXN1*	*ATXN2*	*ATXN3*	*ATXN7*	*CACNA1A*	*HTT*	*JPH3*	*TBP*
Correlation between CAG repeat number and:										
Onset of disease (FTD or ALS)^a^	−0.03 (*0.73*)	−0.01 (*0.88*)	0.02 (*0.75*)	−0.09 (*0.22*)	**0.17** (***0.016***)	−0.01 (*0.88*)	0.04 (*0.63*)	**−0.15 (*0.034*)**	−0.05 (*0.51*)	−0.01 (*0.84*)
Onset of FTD^a^	−0.02 (*0.81*)	−0.04 (*0.60*)	0.01 (*0.85*)	−0.12 (*0.13*)	0.15 (*0.06*)	−0.03 (*0.68*)	−0.04 (*0.63*)	**−0.16 *(0.033)***	−0.05 (*0.56*)	−0.02 (*0.81*)
Onset of ALS^a^	−0.20 (*0.10*)	−0.15 (*0.22*)	0.02 (*0.87*)	−0.12 (*0.33*)	0.13 (*0.27*)	0.11 (*0.39*)	−0.02 (*0.84*)	**−0.24 (*0.049*)**	−0.11 (*0.37*)	−0.03 (*0.78*)
Presence of ALS symptoms at last visit^b^	0.59 (*0.44*)	1.64 (*0.20*)	1.53 (*0.22*)	3.18 (*0.07*)	1.02 (*0.32*)	0.52 (*0.47*)	0.46 (*0.50*)	0.24 (*0.63*)	0.08 (*0.77*)	0.40 (*0.53*)
Comparisons between carriers versus non-carriers of IA^c^:										
Onset of disease (FTD or ALS)^d^			*0.95*					***0.0003***		
Onset of FTD^d^			*0.78*					***0.0004***		
Onset of ALS^d^			*0.51*					***0.046***		
Presence of ALS symptoms at last visit^e^			1.07 (*0.89*)					2.1 (*0.53*)		

Italic numbers indicate *P*-value. Significant results are highlighted in bold.

^a^Pearson *r* (correlation) (*P*-value).

^b^Log-likelihood ratio (logistic regression) (*P*-value).

^c^Only *ATXN1* and *HTT* were analysed due to the absence or too small number of IA carriers for other loci.

^d^ *P*-value (Mann–Whitney U-test).

^e^Odds ratio (*P*-value).

### Impact of polyQ IA on the age at disease onset

In the discovery cohort, the mean age at disease onset was significantly different between *IA-HTT* and *HTT*-normal carriers (*P* = 0.0003), considering both FTD (*P* = 0.0004) or ALS onsets (*P* = 0.046) (Table 3). The mean difference of age at disease onset between *IA-HTT* carriers and non-carriers among *C9orf72* patients was 10.33 ± 2.94 years (mean = 59.42 years for carriers of *HTT*-normal alleles versus 49.09 for carriers of *IA-HTT*s). IA of *ATXN1* were not associated with the age at disease onset (*P* = 0.75). The low number of IA-carriers in the *ATXN2* and *TBP* loci (*n* = 4 each) precluded robust statistical comparisons of means.

These association analyses mentioned above were not corrected for possible confounding factors such as sex, population substructures or known *C9orf72*-disease modifiers as usually performed in genetic association studies. To overcome this statistical limitation, we tested the association of *IA-HTT*s with disease onset using a mixed linear model adjusted for sex, relatedness, *TMEM106B*, *C6orf10* and *SLITRK2* genotypes. In this model, patient’ genotype was defined as homozygous for normal *HTT* alleles or heterozygous for *IA-HTT* (we did not detect individuals homozygous for *IA-HTT*). *IA-HTT*s were associated with the disease onset (*β* = −9.86, *P*_adjusted_ = 0.0022). The association was observed with the onset of FTD (*β* = −9.46, *P*_adjusted_ = 0.0031), and with the onset of ALS (*β* = −12.97, *P*_adjusted_ = 0.0101). Altogether, our results suggest that the observed association between *IA-HTT*s and an earlier disease onset persists after adjustments and is independent of known disease modifiers.

### Replication of the association between IA of *HTT* and the age at disease onset

We compared the mean disease onset between *HTT*-normal and *IA-HTT* carriers in an independent cohort of European unrelated *C9orf72* patients (*n* = 145, Fig. 1B). *IA-HTT*s were associated with an earlier disease onset (*P* = 0.0059). The difference of age at onset between *IA-HTT* carriers and non-carriers was 8.36 ± 3.14 years. As expected, the analysis in the merged population (*n* = 340) reinforced the statistical association of *IA-HTT*s with an earlier disease onset among *C9orf72* patients (*P* = 1.3 × 10^e−5^, Fig. 1C) with a mean difference of 9.42 ± 2.14 years between *IA-HTT* carriers and non-carriers.

### Detection of potential somatic expansions of CAG in *IA-HTT* carriers

**Figure 2:**
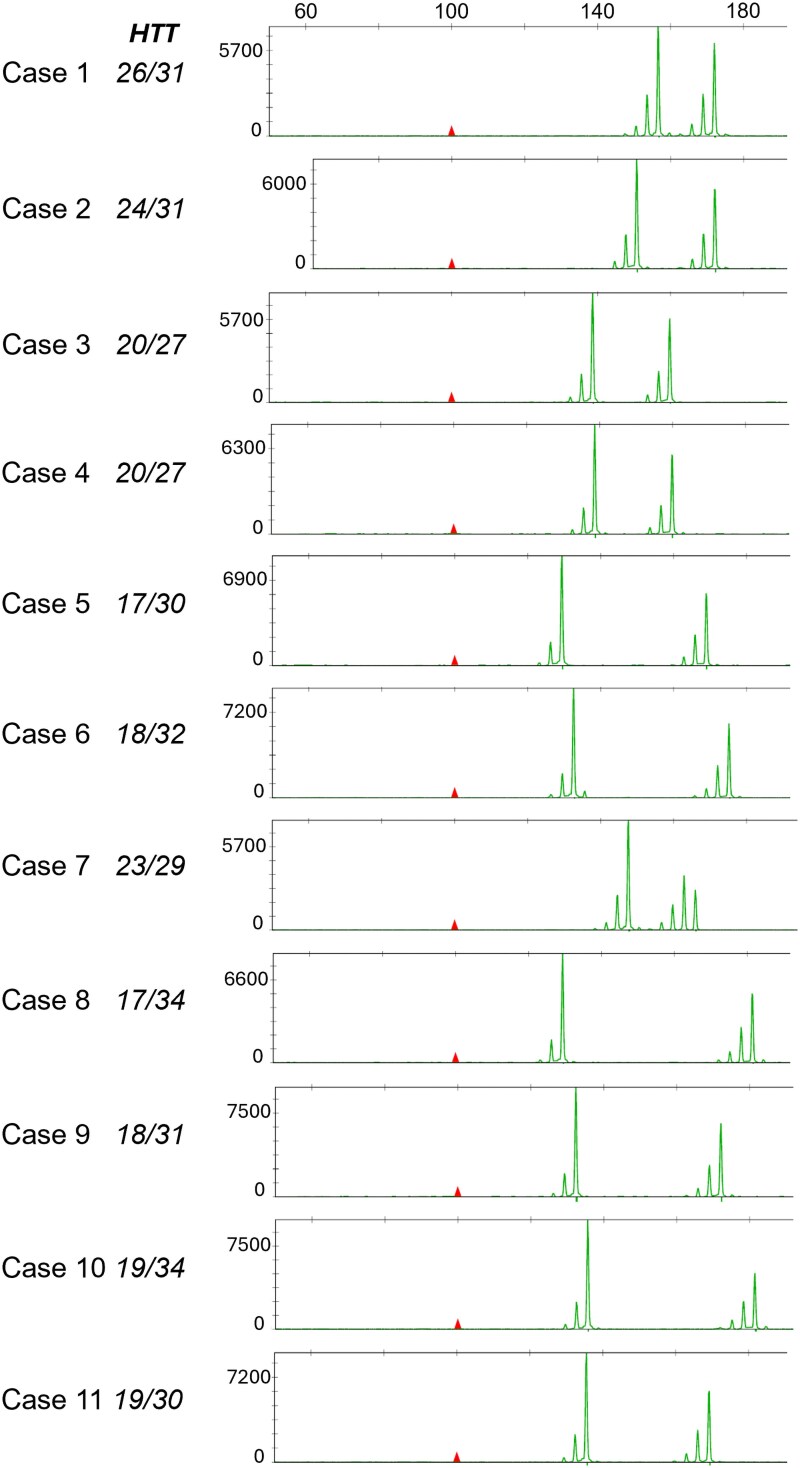
***HTT* CAG repeat profiles in *C9orf72* patients’ blood carrying *IA-HTT*s from PCR-fragment analyses.** X-axis, top of the figure: PCR length (bp, same scale for all profiles), Y-axis: fluorescent units. Genotypes inferred from these profiles are indicated and in accordance with estimates from short-read whole-genome sequencing.

We analysed the potential presence of somatic expansion in *IA-HTT* carriers. We first looked in aligned reads from the whole-genome sequencing and did not detect reads with more CAGn than the maximum number estimated (data not shown). Second, the careful examination of PCR profiles from the fragment analyses in blood DNA of *C9orf72exp*/*IA-HTT* carriers was not in favour of somatic expansions (Fig. 2). We had access to brain tissues of one *C9orf72exp*/*IA-HTT* carrier (Case 1, Fig. 2). Unfortunately, the weak quality of DNA extracted from paraffin blocks (no frozen tissue available) precluded a reliable detection of somatic variations of *HTT* CAG repeats.

### Clinical characteristics of *C9orf72* patients with *IA-HTT*

In the merged cohort, 5.8% were heterozygous carriers of *IA-HTT*s with 27 to 34 CAG. All patients developed FTD symptoms, from 30 to 65 years (mean disease onset = 49.67 ± 1.75 years). Six patients also developed symptoms of ALS diagnosed at last visit. Globally, except for the early disease onset, we did not observe clinical particularity between these *C9orf72* patients with *IA-HTT*s and typical *C9orf72* cases. Nonetheless, three of them showed a disease duration at death remarkably longer than usually observed (18, 20 and 24 years, respectively).

### Neuropathology of a case carrying both *C9orf72exp* and *IA-HTT*

**Figure 3:**
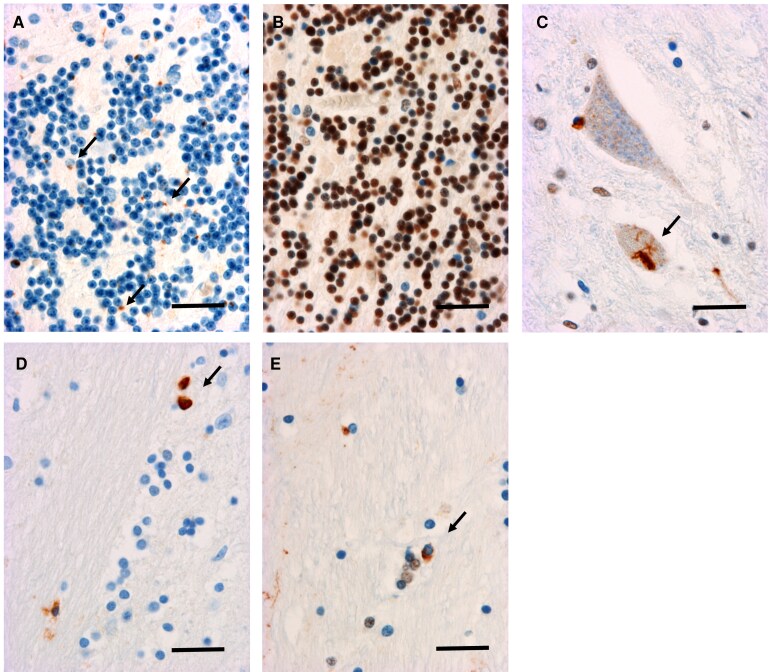
**Neuropathology of a *C9orf72* case carrying one intermediate allele of *HTT* (CAG_n_ = 26/31).** (**A**, **B**) Cerebellar grain layer showing discrepancy between the presence of p62+ inclusions (**A**, arrows) and the absence of loss of normal TDP43+ labelling of nuclei (**B**); (**C**) Skein-like inclusions in motor neurons of the anterior horn of the spinal cord. (**D**, **E**) Striatum. Oligodendroglial inclusions seen with anti-p62 (**D**, arrows) and anti-TDP43 (**E**, arrows). Scale bars = 20 μm.

**Figure 4:**
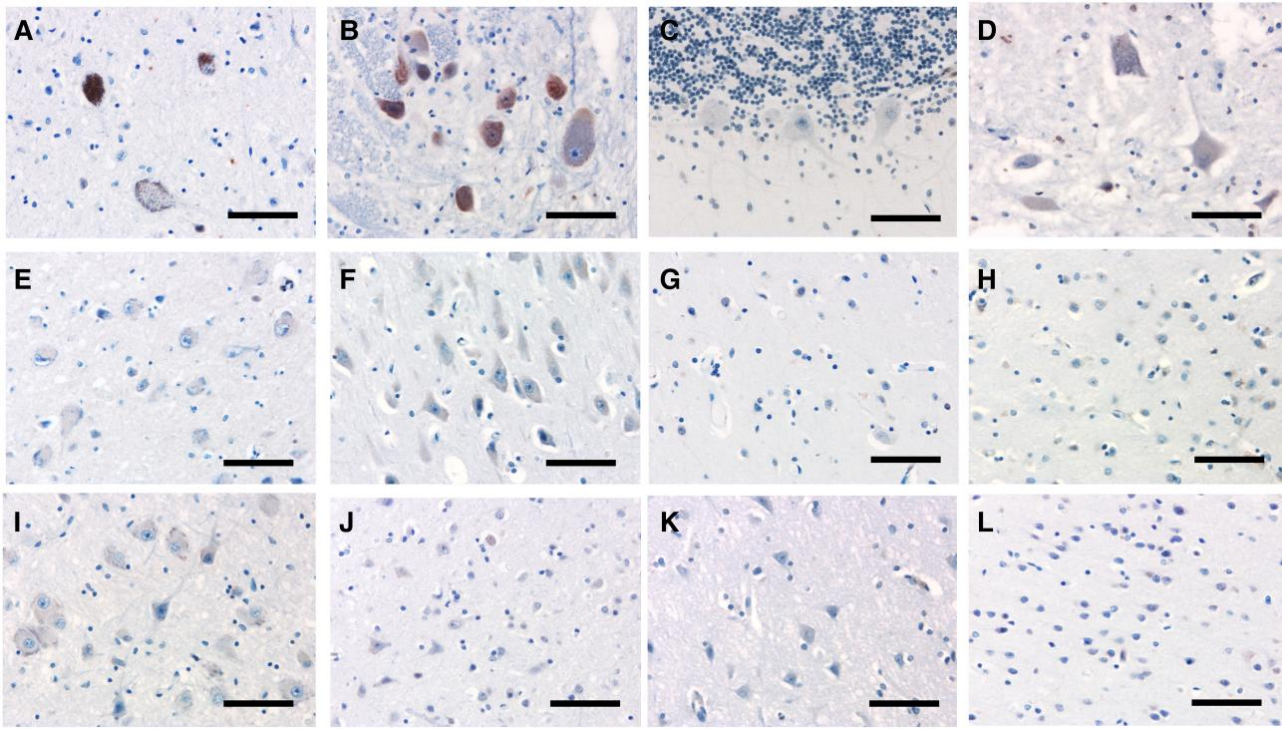
**Anti-HTT immunohistochemistry in twelve regions of the central nervous system of the patient carrying *C9orf72exp* and *IA-HTT*.** (**A**) lower mesencephalon; (**B**) inferior olivary nucleus; (**C**) cerebellum; (**D**) cervical spinal cord; (**E**) thalamus; (**F**) hippocampus; (**G**) putamen; (**H**) caudate nucleus; (**I**) nucleus basalis of Meynert; (**J**) midfrontal cortex; (**K**) temporal pole; (**L**) calcarine sulcus. Diffuse intracytoplasmic positivity were observed in the inferior olivary olive (**B**). No intranuclear inclusions were found in any region examined. Scale bars = 40 μm.

A neuropathological analysis was performed in a *C9orf72* patient carrying *IA-HTT* (Figs 3 and 4). The patient (male) presented an early onset of FTD at 48 years, then he developed first symptoms of ALS (bulbar onset) 3 years later (51). He died at the age of 52 years. The sequencing data revealed a canonical structure of polyglutamine repeats without duplication or loss of the CAACAG motif. Other alleles for polyglutamine loci were in the normal range. The lesions observed were intracytoplasmic TDP-43- and p62-positive inclusions in the associative cortices and dentate gyrus of the hippocampus (Fig. 3). In the cerebellum, there was a discrepancy between p62-positive and TDP-43-negative labelling. In the spinal cord and medulla oblongata, there were neuronal intracytoplasmic inclusions suggestive of ALS lesions in line with the clinical picture of this patient (FTD-ALS). Overall, the appearance was that usually encountered in *C9orf72* mutations. Immunohistochemistry for 1C2, labelling the aggregation of polyglutamine stretches observed in polyQ diseases such HD, was negative in all regions examined including frontal, temporal, parietal, occipital cortices, hippocampus, medulla oblongata, spinal cord, thalamus, anterior hippocampus, basal nucleus of Meynert, calcarine and striatum, and positive (intranuclear) for the HD case (Supplementary Fig. 2). In addition, we performed specific immunostaining of HTT in the same 12 regions (Fig. 4). A diffuse punctate cytoplasmic staining was variably observed in both *C9orf72*/*IA-HTT* case and in the negative control. This immunoreactivity is similar to what has been described in non-HD cases using this antibody.^25^ Intranuclear inclusions were only observed in the positive control (HD case with 40 CAG in *HTT*). No intranuclear inclusion of HTT could be observed in the *C9orf72* case with *IA-HTT* (Fig. 4).

## Discussion

Genetic modifiers contributing to the age of onset and clinical variability in *C9orf72*-related disease remain largely unknown. In previous studies, the search for genetic modifiers in *C9orf72* disease, as well as in many other diseases, has been mainly assessed using SNP-based genome-wide association studies which poorly tag potential signals of association coming from multi-allelic STRs.^29^ This is a significant limitation, particularly in studying genetic modifiers in FTD-ALS, given the well-established molecular and biological links between TDP-43 aggregation pathologies and diseases associated with CAG polyQ expansions. In this work, we conducted whole-genome sequencing to overcome this bias and evaluated the contribution of polyQ associated-STRs as potential modifiers of *C9orf72* disease.

*IA-HTT*s were associated with an earlier onset of *C9orf72* disease. This association was observed after performing a statistical model adjusted for potential confounding biases and was replicated in an independent European cohort. Previous studies pointed-out the haplotypic and allelic diversity linked to *IA-HTT*s and suggested that *IA-HTT*s cannot be perfectly tagged by bi-allelic SNPs traditionally used in genome-wide association studies.^30,31^ This could explain why SNP-based genome-wide association studies previously published and carried out on *C9orf72* cohorts failed to detect a signal at this locus.

Our results reinforce previous works suggesting that the size of polyQ associated STRs influences disease outcomes in TDP-43 associated diseases.^10,11,19,32^ More recently, *IA-HTT*s have been shown to influence the course of the disease in non-genetic forms of ALS.^33^ Our results suggest that *IA-HTT* are also modifiers of *C9orf72* FTD/ALS. The association between *IA-HTT*s and *C9orf72* phenotypes is reminiscent of the association between *ATXN2*-IAs and the risk to develop ALS, replicated in *C9orf72* cohorts and also detected in this work.^9,11^

*ATXN2* modifies the TDP-43 toxicity in animal and cellular models, possibly by promoting the abnormal cytoplasmic localization of TDP-43.^9^ This hypothesis was reinforced in a more recent study describing how the methyl 1 adenosine (m^1^A)/non methylated adenosine ratio in CAG repeat RNA increases with repeat length, and how TDP-43 can interact with m1A in CAG repeat to promote the cytoplasmic mislocalization of TDP-43.^8^ This pathological mechanism could be common to all polyQ loci. However, in our study and in the literature, not all polyQ loci were associated with clinical traits possibly because of specific regional patterns of expression of polyQ genes, and/or specific interaction with C9orf72. Nevertheless, all the studies mentioned above highlighted that CAG tracks perturb the cellular TDP-43 behaviour which could be a mechanism explaining the adverse effect of IA.

Indeed, the pathological hallmarks of *C9orf72* and HD, TDP-43 and PolyQ aggregates respectively, can coexist in brain of HD patients with pathological *HTT* CAG repeat numbers and in vitro analyses have shown that mutant huntingtin (mHTT) favours the seeding properties of TDP-43.^4,5,34^ The neuropathological examination of one *C9orf72* patient carrying *IA-HTT* revealed a typical picture of *C9orf72* disease. The presence of polyglutamine inclusions in cerebral tissue from *IA-HTT* carriers is very discussed, with variable results across studies. So far, two other papers described neuropathological examinations of carriers with *IA-HTT* (27–35 CAG) and immunostainings for polyQ.^35,36^ Among the six patients carrying *IA-HTT* (all with comorbidities), two were positive for polyQ inclusions, all carrying 32 CAG repeats. The four remaining patients carrying from 27 to 33 CAG for the longest allele in *HTT* and described in these two studies were negative for polyQ inclusion. A third study specifically focused on the HTT protein in a series of three patients, one carrying *IA-HTT* (33 CAG). Few extra-nuclear inclusions of HTT in the frontal cortex and pons were detected in this patient.^37^ Thus, the synthesis of these works does not allow us to draw definite conclusions on the genotype–phenotype correlation between *IA-HTT* and the presence of polyQ or HTT inclusions. These differences could come from the immunostaining varying between HTT or polyQ and regions tested. We performed a staining for polyQ as well as a specific staining for HTT in brain tissue from the *C9orf72*/*IA-HTT* case carrying 31 CAG. No inclusion could be observed in all regions tested.

Therefore, the neuropathological case considered in our study seems to exclude the coexistence of TDP-43 and polyQ or HTT aggregates as the pathological mechanism to explain the greater pathogenicity of *IA-HTT*s in *C9orf72* carriers. Additional cases carrying both *C9orf72*exp and *IA-HTT* are necessary to draw definite conclusions about neuropathological findings described in this study. However, it’s interesting to note that despite the scarcity of such report, inclusions were variably observed in patients with *IA-HTT* ≥ 32 CAG, and never with a smaller number of repeats. It therefore possible that a threshold around 32/33 CAG could explain the determinism of these inclusions, depending also on other co-variables such as the brain regions analysed, age at death or existing co-pathologies.

Also discussed is the instability of *IA-HTT*, more prone to somatic expansions. No clear somatic expansion of *IA-HTT* has been detected in blood. Unfortunately, the corresponding analysis was not feasible in the brain of the *C9orf72* patient also carrying *IA-HTT*. Thus, we cannot rule out that somatic expansion of *IA-HTT*s may happen in patients’ brains that could worsen the *C9orf72* disease as somatic expansion is a mechanism known to be highly tissue-specific. However, the absence of somatic expansion in blood of *IA-HTT*s is in line with another recent study which notably showed the absence of somatic expansion of *IA-HTT* in different brain regions of patients with tauopathies.^38^

All the hypotheses cited above only imply *HTT* or more globally CAG-linked mechanisms. One can suppose that *IA-HTT*s worsen biological pathways already affected in the *C9orf72* disease. Indeed, both pathogenic expansions in *C9orf72* and *HTT* have been shown to affect pathways such as axonal and nucleocytoplasmic transports as well as DNA damage.^39-42^ A more in-depth analysis of these biological functions in cellular models may offer the opportunity to detect which pathway can influence the age at disease onset. Another potential candidate pathway linked to *HTT* is the BDNF/TrkB pathway. One of the first known physiological roles of HTT is the transport of BDNF-containing vesicles.^42^ Interestingly, *SLITRK2*, one of the few known genetic modifiers of the age at onset in *C9orf72*-disease, has also been linked to BDNF/TrkB signalling.^14,43^ The hypothesis that *IA-HTT*s could alter this pathway already affected in the context of the *C9orf72* expansion also remains to be tested.

Overall, our results illustrate the growing interest in considering STRs as disease modifiers, although this type of dynamic polymorphism has been clearly neglected in almost all previous genetic association studies. Our study only included polyQ loci which is a conceptual limitation, however justified by existing biological links between *C9orf72* and polyQ diseases, and by the reliability of genotyping these specific STRs from short-read sequencing. Genome-wide and without a priori approaches to include STRs may allow the discovery of new modifying loci that could have been missed before. Incorporating polymorphic and potentially functional STRs in association studies will reveal more precisely the genetic architecture genetic modifiers in FTD/ALS.

## Supplementary Material

fcaf220_Supplementary_Data

## Data Availability

Raw sequencing data were generated at iGenSeq core facility. Derived data supporting the findings of this study are available from the corresponding author on request. All methods and pipelines of analyses used in this study are already deposited in public repositories.

## Appendix

**The French clinical and genetic research network on FTLD/FTLD-ALS and PrevDemALS groups include:**

Alexis Brice, Sophie Auriacombe, Serge Belliard, Frédéric Blanc, Stéphanie Bombois, Claire Boutoleau-Bretonnière, Agnès Camuzat, Mathieu Ceccaldi, Philippe Couratier, Vincent Deramecourt, Mira Didic, Frédérique Etcharry-Bouyx, Maïté Formaglio, Véronique Golfier, Didier Hannequin, Lucette Lacomblez, Julien Lagarde, Isabelle Le Ber, Richard Levy, Florence Pasquier, Thibaud Lebouvier, Carole Roué-Jagot, Anne Salmon, Marie Sarazin, Christel Thauvin-Robinet, Catherine Thomas-Anterion, Jérémie Pariente, François Sellal, Daisy Rinaldi, Adeline Rollin-Sillaire, Martine Vercelletto and David Wallon.
