# Supplementary material for: Analysis of short tandem repeats linked to polyglutamine diseases from whole-genome sequencing reveals intermediate alleles of HTT associated with an early disease onset in C9orf72 carriers
Source: Brain Commun. 2025 Jun 4;7(3):fcaf220. doi: 10.1093/braincomms/fcaf220 (PMC12204198; doi:10.1093/braincomms/fcaf220)
Supplement: fcaf220_Supplementary_Data [file fcaf220_supplementary_data.docx]

**Supplementary Material**

**TITLE**

**Analysis of short tandem repeats linked to polyglutamine diseases from whole-genome sequencing reveals intermediate alleles of *HTT* associated with an early disease onset in *C9orf72* carriers.**

**AUTHORS AND AFFILIATIONS**

Mathieu Barbier, Thomas Gareau, Agnès Camuzat, Marine Guillaud-Bataille, Susana Boluda, Fabienne Clot, Lara Araktingi, **Barbara Borroni^,^, Julie van der Zee, Roberta Ghidoni, Sonia Bellini, Daniela Galimberti, Giacomina Rossi, Benedetta Nacmias, Beatriz De la Casa-Fages, Pau Pastor, French clinical and genetic research network on FTD/FTD-ALS and PrevDemALS study groups,** Morwena Latouche, Eric le Guern, Alexandra Durr, Annie Laquerrière, Rob Moccia, Danielle Seilhean, Victoria Alvarez, and Isabelle Le Ber.

**Supplementary Figure 1. Measure of *HTT* CAG repeat number.** Comparison between estimates from whole-genome sequencing data and PCR-fragment length analyses. Results for short (**A**) and long (**B**) alleles appear separately for each patient (22 alleles tested).


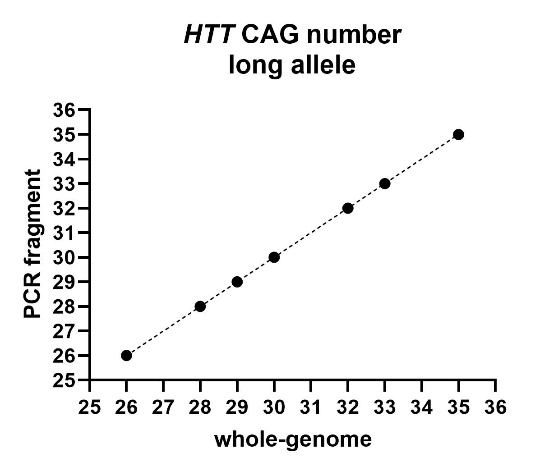

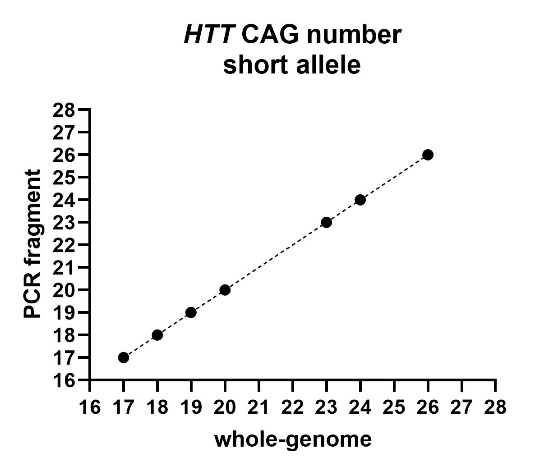


**B**

**A**

**Supplementary Figure 2. Immunostaining for 1C2 (A-C) and huntingtin (D-F) in the head of the caudate nucleus in a Huntington control (A,D), the case carrying both *C9orf72exp* and *IA-HTT* (B, E), a control case without any neurological condition (C,F).** Punctiform intranuclear inclusions are observed only in the Huntington case with both antibodies (**A, D,** large arrows), while Huntingtin + intracytoplasmic labeling was observed not only in the Huntington case but also in the negative control (**D, F**, thin arrows). Scale bars = 20 μm.


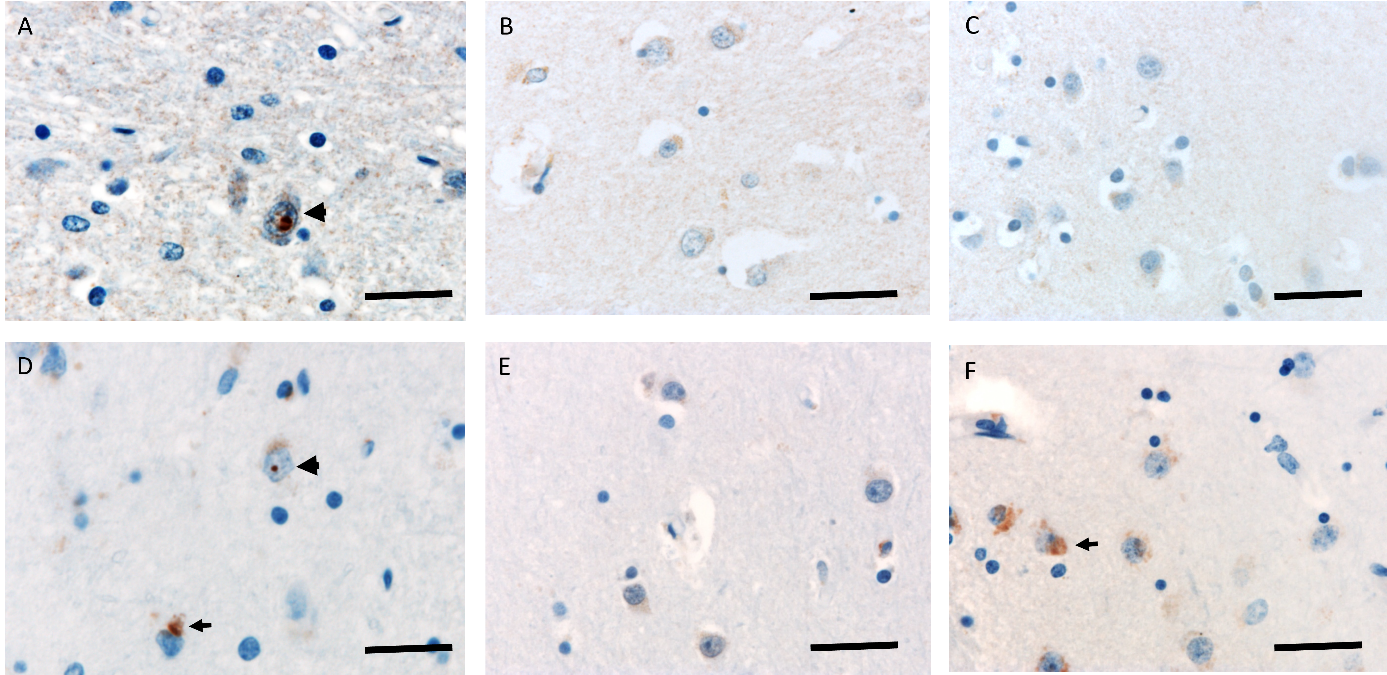


**Supplementary Table 1. Immunohistochemical methods.**

| **Antigen** | **Poly/monoclonal** | **Producer** | **Immunogen** | **Clone** | **Pretreatment** | **Dilution** | **Incubation**  **time** |
| --- | --- | --- | --- | --- | --- | --- | --- |
| Huntingtin | Mouse monoclonal | Merck® | N-terminal huntingtin | 2B4 | CC1®  64 min | 1:250 | 1h00 |
| Polyglutamine | Mouse monoclonal | Euromedex® | Polyglutamine repeats | 1C2 | Semi-manual, peroxidase and biotin blocking | 1 :3000 | 48h00 |
| p62 | Mouse monoclonal | BD Biosciences® | p62-lck ligand | 3/p62 | CC2®  44 min | 1:500 | 1h36 |
| pTDP43 | Mouse monoclonal | Cosmo Bio® | CMDSKS(p)S(p)GWGM- pS409/410-2 | 11-9 | CC1®  8 min | 1 :5000 | 1h00 (amplified) |
| TDP43 | Rabbit polyclonal | Proteintech® | N-terminal  TDP-43 | _ | CC1®  8 min | 1 :2000 | 1h20 |
| Ubiquitin | Rabbit polyclonal | Dako® | Ubiquitin (cow erythrocytes) | _ | CC1®  8 min | 1 :500 | 32 min |

**Supplementary Table 2. Descriptive data of the positive control (Huntington disease, HD case) used for neuropathological analyses.**

|  | HD (2569-005) |
| --- | --- |
| Sex/age at onset in years/age at death in years | Man/80/90 |
| CAG repeat in first exon of *HTT* (pathological) | 40 |
| Last examination (Unified Huntington Disease Rating Scale/124) | 36 at age 86 years (0 at age 79) |
